# Pharmaceutical residues in food samples: occurrence, toxicity, fluorescence quenching mechanisms, and analysis using carbon dot-based fluorescent probes

**DOI:** 10.1039/d6ra08144d

**Published:** 2026-10-13

**Authors:** Kawan F. Kayani

**Affiliations:** a Department of Chemistry, College of Science, University of Sulaimani, Kurdistan Regional Government Qlyasan Street Sulaymaniyah 46001 Iraq kawan.nasralddin@univsul.edu.iq

## Abstract

Carbon dots (CDs) are an important class of carbon-based nanomaterials and represent a relatively new type of fluorescent nanomaterial that has attracted extensive research interest. Their popularity has increased rapidly due to several advantageous characteristics, including low toxicity, chemical stability, tunable fluorescence behavior, good water solubility, and favorable physicochemical properties. Owing to these features, CDs have been widely explored as sensing materials for the detection of various targets. In this review, pharmaceutical residues in food samples and their associated toxicity are discussed, along with the fluorescence sensing mechanisms and applications of CD-based sensors for the detection of a lot of pharmaceutical drugs in food samples, including tetracyclines, chloramphenicol, ciprofloxacin, gentamicin, sulfonamides, metronidazole, moxifloxacin, penicillin and others. Finally, the future prospects of using CDs for pharmaceutical analysis in food samples are also reviewed.

## Abstract 2

In this review, pharmaceutical residues in food samples and their associated toxicity are discussed, along with the fluorescence sensing mechanisms and applications of CD-based sensors for detecting various pharmaceutical drugs in food samples.

## Graphical abstract

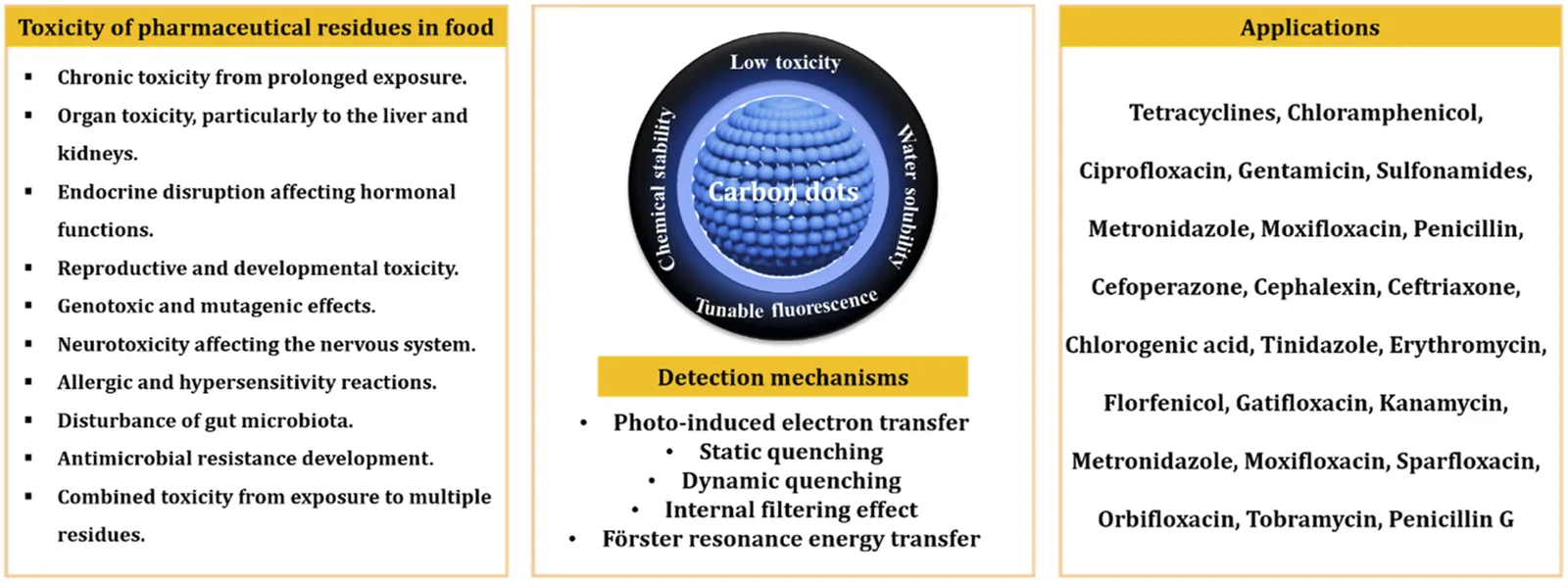

## 1. Introduction

With the rapid growth of the global population and economy, maintaining food safety has become an increasing challenge for the food industry.^1^ Expanding industrial activities and intensive agricultural practices, although enhancing food production, have also contributed to the introduction of contaminants, adulterants, and biological hazards such as fungi and bacteria into the food chain.^2^ As a result, food analysis has become a critical and necessary task to ensure the safety and quality of food supplies in an expanding world.^3^ Various contaminants, including those arising from adulteration, microbial deterioration, or other sources, can pose serious risks to consumers.^2,4^

Pharmaceutical compounds are considered a significant group of emerging environmental contaminants.^5,6^ Residues of these substances have been identified in various environmental compartments around the world, including surface waters, wastewater, sediments, and sludge. Depending on their hydrophilic properties, these compounds may either dissolve and disperse in aquatic systems or become attached to particulate matter.^7^ Major contributors to their environmental presence include domestic sources, wastewater treatment facilities, hospitals, pharmaceutical manufacturing industries, and intensive livestock farming operations. A major concern is that the continuous exposure of aquatic environments to low concentrations of antibiotic residues can promote the development of antibiotic-resistant bacterial strains.^8,9^

Pharmaceutical and veterinary drug residues are important contaminants of food and feed, arising from the use of antibiotics, antiparasitic agents, growth promoters, and other therapeutic compounds in livestock and aquaculture. These substances may remain as parent compounds or metabolites in products such as meat, eggs, fish, poultry, and ready-to-eat foods.^10^ Their occurrence has raised increasing concerns because prolonged exposure to such residues may contribute to antimicrobial resistance, allergic reactions, endocrine disturbances, and other potential health effects. In addition, these compounds can persist in the environment, accumulate in aquatic and terrestrial ecosystems, and potentially transfer through the food chain.^11^ Therefore, monitoring pharmaceutical residues in food and the environment is important for ensuring food safety, protecting public health, and minimizing ecological risks.

According to the European Food Safety Authority (EFSA), drug residues are described as pharmacologically active compounds, along with their excipients, metabolites, and degradation products that remain present in animals, food products, or environmental systems. These residues can occur in foods of animal origin, particularly when veterinary drugs are improperly used or when recommended withdrawal periods are not followed.^11^ Accurate determination of pharmaceutical compounds, particularly in real samples, is analytically challenging due to the complex nature of the sample matrices and the typically low concentrations at which these substances are present.^12,13^

Currently, a variety of techniques have been developed for the detection of pharmaceutical residues including chromatographic methods,^14^ electrochemical,^15^ surface-enhanced Raman spectroscopy,^16^ and enzyme-linked immunosorbent assays (ELISA).^17^ However, many of these techniques are limited by high costs and operational complexity. In contrast, optical sensing systems offer significant promise for detection applications because they are simple to use, provide rapid responses, and exhibit high sensitivity.^18^ Luminescence-based sensing technologies have attracted considerable attention due to their ability to provide real-time, non-invasive, and visual detection of analytes.^19^ Among these approaches, fluorescence methods have received significant interest because they are cost-effective, highly sensitive, enable rapid analysis, and are simple to operate.^20–22^

Typical fluorescent nanomaterials comprise organic dyes, quantum dots, and metal nanoclusters materials. Nevertheless, their application in food analysis is often constrained by drawbacks such as photobleaching and toxicity.^23^ Consequently, there is an increasing demand for new fluorescent nanomaterials that provide enhanced performance while ensuring safety. Carbon dots are part of the broader class of carbon-based nanomaterials and are considered an important member because of their unique fluorescent properties.^24^ It is noteworthy that CDs were first observed inadvertently during the purification of single-walled carbon nanotubes (SWCNTs) produced by the arc-discharge technique.^25^ Furthermore, in 2006, Ya-Ping *et al.* formally introduced the term “carbon dots” to describe this new type of material.^26^ Compared to conventional fluorescent materials, CDs offer several advantages, including small sizes below 10 nm and high oxygen content. Owing to quantum confinement and edge effects, they also exhibit distinctive properties such as tunable emission, excitation-wavelength dependent photoluminescence (PL), excellent water solubility, and remarkable photostability.^27–37^ These advantages make CDs attractive nanomaterials for a wide range of applications, particularly as fluorescent nanoprobes for the detection of pharmaceutical residues.

Despite several published reviews on CDs, pharmaceutical analysis in food samples has become one of the most attractive research fields in recent years. However, to the best of our knowledge, no comprehensive review summarizing all relevant articles on pharmaceutical analysis in real food samples has yet been reported. Therefore, this review is unique and provides a comprehensive overview of this topic. The review discusses the toxicity of pharmaceutical residues in food samples, the underlying sensing mechanisms, and the applications of CDs for the detection of a wide range of pharmaceutical residues. Finally, the conclusions and future perspectives are also presented.

### 1.1 Comparison of the present review with previous studies

Despite the numerous reviews published on carbon dot-based sensing applications,^38–43^ to the best of our knowledge, no comprehensive review has yet focused specifically on the detection of pharmaceutical residues in food samples using CDs. Therefore, the present review is unique and timely. It comprehensively covers relevant studies on CDs for the sensing of pharmaceutical residues in food samples. The occurrence and potential toxicity of these residues are first discussed, followed by a systematic classification of the fluorescence quenching mechanisms involved in carbon dot-based sensing. Subsequently, the relevant studies are critically discussed in the application section and categorized into appropriate subsections. Finally, the review provides conclusions and future perspectives on the development of novel carbon dot-based fluorescent sensors for the sensitive and selective detection of pharmaceutical residues in food samples.

## 2. Pharmaceutical residues in food samples and their toxicity

The occurrence of pharmaceutical residues in food is a significant concern because of their potential adverse effects on human health. These residues mainly originate from veterinary medications that are commonly applied in livestock production. Such drugs are administered to food-producing animals to prevent and treat diseases, promote growth, and enhance feed efficiency. As a result, traces of these compounds, either as the original substances or as their metabolites, may remain in food products. In some cases, these residues can accumulate and become amplified along the food chain, posing health risks to humans when their concentrations exceed acceptable limits.^10^

Pharmaceutical residues in food samples particularly veterinary drugs such as antibiotics used in animal production pose significant toxicological risks to human health even at low concentrations. These residues may remain in meat, milk, honey, eggs, and other food products when drugs are misused or when withdrawal periods are not respected.^44^ Chronic exposure can lead to several adverse effects, including allergic and hypersensitivity reactions, disruption of the normal intestinal microbiota, and direct toxic effects on organs such as the liver, heart, kidneys, and bone marrow.^44,45^ In addition, some pharmaceutical residues or their metabolites may exhibit mutagenic, teratogenic, or carcinogenic potential, and certain compounds have been associated with reproductive disorders and nephrotoxicity.^46^ However, the most critical public health concern is the development and spread of antibiotic-resistant bacteria due to continuous low-dose exposure through contaminated food, which reduces the effectiveness of therapeutic antibiotics and threatens global health security.^47,48^ Moreover, drug toxicity in food and the environment can occur due to excessive use, as these compounds can be distributed through the bloodstream and accumulate in various organs. Consequently, adverse drug reactions (ADRs) and toxicity cannot be ignored, and the U.S. Food and Drug Administration must take regulatory action when necessary. For example, the agency has previously withdrawn the medication mipomersen from the market due to concerns about hepatotoxic effects.^49^ Therefore, monitoring and the sensitive detection of pharmaceutical residues in food samples are essential to ensure food safety and protect human health.

## 3. Fluorescence quenching mechanisms

### 3.1 Photo-induced electron transfer (PET)

PET is a process in which the excited state of a fluorophore, such as CDs, interacts with another species capable of donating or accepting an electron. During PET, an electron donor–acceptor complex forms, which can return to the ground state without emitting light. In some cases, emission from the exciplex may occur. Eventually, the electron is transferred back to the donor.^40,50^ The synthesized nitrogen- and sulfur-doped carbon dots (NS-CDs) exhibited excellent photoluminescence stability and a high quantum yield (QY) of 91.55%. The fluorescence intensity of the NSCDs was effectively quenched upon the addition of tetracycline (TC), mainly through the inner filter effect (IFE) and PET mechanisms. Accordingly, the NS-CDs were successfully employed as a simple and sensitive fluorescent probe for TC detection over a linear concentration range of 0.2–70 µM, with a low detection limit of 0.072 µM. The recovery studies were conducted using the standard addition method, with recoveries ranging from 96.5% to 108% in real samples of water, milk, and honey.^51^ Pan *et al.* developed portable carbon dot-based fluorescent molecularly imprinted photonic crystal hydrogel strips for TC detection in animal-derived foods. The sensor showed a linear range of 0.1–50 µg mL^−1^ and an LOD of 0.067 µg mL^−1^. Electron transfer from the CDs to the lowest unoccupied molecular orbital (LUMO) of TC leads to fluorescence quenching, thereby confirming the PET mechanism. The accuracy of the proposed method was evaluated using the standard addition technique, yielding recovery values of 93.6–112.59% for the analysis of animal-derived foods pork, eggs, and milk samples.^52^

### 3.2 Static quenching and dynamic quenching

Static quenching occurs when a non-fluorescent complex forms between CDs and a quencher in the ground state. Upon light absorption, this complex quickly relaxes to the ground state without photon emission. Characteristics of static quenching include: (1) possible changes in the absorption spectrum due to complex formation, (2) increased fluorescence with temperature as weakly bound quenchers dissociate, and (3) unchanged fluorescence lifetimes of CDs. Due to its easily verifiable mechanism, static quenching is widely applied for detecting analytes.^53^ Dynamic quenching arises from collisions between analytes and excited-state CDs, which reduce fluorescence without affecting the absorption spectrum. This process relies on analyte diffusion and occurs within the fluorophore's excited-state lifetime.^54^ Dual-emission lanthanum-doped carbon dots (La-CDs) have been developed for the simultaneous detection of oxytetracycline (OTC) and tetracycline (TC). The sensor exhibited linear response ranges of 0.00–805.20 µM for OTC and 0.00–1039.50 µM for TC. The sensing mechanism was concentration-dependent. At low OTC concentrations, the fluorescence quenching was mainly attributed to the IFE and static quenching. At higher OTC concentrations, the IFE and dynamic quenching mechanisms coexisted with static quenching. The recovery studies were performed in actual samples of pork and fish with satisfactory results.^55^ CDs have been developed for the detection of TC, OTC, and chlortetracycline (CTC), with linear ranges of 0.5–30.0 µM, 0.4–15.0 µM, and 0.5–10.0 µM, respectively. The fluorescence quenching of the CDs by the tetracyclines was attributed to a combination of the IFE and static quenching, with static quenching considered the predominant mechanism. Recovery studies were performed using real samples of milk and pig liver.^56^

### 3.3 Internal filtering effect (IFE)

IFE occurs when an absorber reduces fluorescence by capturing excitation or emission light from the fluorophore. Unlike FRET, IFE avoids complex labeling, offering high sensitivity, selectivity, and simplicity. The performance of an IFE-based sensor depends on the spectral properties and overlap of the absorber and fluorophore.^57–59^ Through the IFE, TC has been detected in milk samples using CDs. The fluorescence intensity of the CDs was enhanced by incorporating graphene, and the analytical performance included a limit of detection (LOD) of 0.9284 nM. The spiked recovery study using the standard addition method was performed to determine TC in real samples, with recovery values ranging from 99.77% to 101.38%.^60^ Another fluorescent sensor for CTC based on N,S-doped carbon dots (N,S-CDs) has also been developed. The sensor exhibited a linear range of 1.24–165 µM and an LOD of 32.36 nM. The sensing mechanism was attributed to the IFE, because the UV-vis absorption spectrum of CTC significantly overlapped with the fluorescence excitation spectrum of the N,S-CDs. Moreover, the fluorescence lifetime did not change significantly after the addition of CTC, further supporting the IFE-based sensing mechanism. The proposed sensor was applied to water and milk samples. The original concentration of CTC in the unspiked samples was found to be zero, indicating that CTC was not naturally present at detectable levels. Therefore, only spiked samples were used for further analysis.^61^ Lastly, nitrogen-doped carbon dots (N-CDs) were synthesized using pomelo peel as a carbon precursor for the detection of TC. The resulting N-CDs exhibited a linear response toward TC over the concentration range of 0–100 mmol L^−1^, with a detection limit of 0.045 mmol L^−1^. The fluorescence lifetime measurements further supported the proposed sensing mechanism. In the absence of TC, the average fluorescence lifetime of the N-CDs was 8.01 ns, while it decreased slightly to 7.96 ns after the addition of TC. Since this negligible change in fluorescence lifetime indicates that no significant dynamic quenching occurred, the fluorescence response was attributed to the IFE. In this work, milk was selected as a real sample, and the spiked recovery values ranged from 98.8% to 102.7%.^62^

### 3.4 Förster resonance energy transfer (FRET)

FRET is one of the most commonly used quenching mechanisms for CDs. Energy transfers from a donor fluorophore to an acceptor chromophore through dipole–dipole interactions without photon emission. The efficiency of FRET depends on dipole orientation, spectral overlap, and donor–acceptor distance, typically between 1 and 10 nm. Depending on the acceptor's properties, the energy can either generate fluorescence or dissipate as heat.^63^ Blue-emitting CDs were prepared from glucose and used for the detection of TC in food samples, achieving a limit of detection (LOD) of 31.1 nM. According to the reported literature, the sensing mechanism was attributed to FRET. Honey was used as a real sample for the determination of TC, with spiked recovery values ranging from 90.31% to 109.48%.^64^ Blue-emitting ZrN-CDs were synthesized hydrothermally from citric acid, zirconium chloride, and ethylenediamine. A dual-emission ratiometric sensor was developed by combining ZrN-CDs with Eu^3+^, where tetracyclines quenched the CD fluorescence through FRET while enhancing Eu^3+^ emission *via* the antenna effect. Using the intensity ratio, TET and OTC were detected with LODs of 0.029 and 0.016 µM, respectively. In this work, spiked recovery studies were performed to evaluate the reproducibility of the proposed sensor. Different types of milk were selected as real samples, and the recovery values ranged from 90.91% to 113.19%.^65^

Fluorescence lifetime spectroscopy is an important analytical technique that offers valuable information about molecular dynamics and interactions by determining the time a molecule remains in an excited state following light absorption.^66^ The fluorescence lifetime generally refers to the average duration that a molecule spends in the excited state before returning to its ground state.^67^ In sensing applications, a reduction in fluorescence lifetime upon interaction with an analyte is typically indicative of a dynamic quenching mechanism. Conversely, when the fluorescence lifetime remains essentially constant despite a decrease in fluorescence intensity, the quenching is commonly associated with the IFE or static quenching mechanisms, as demonstrated in several studies.^68^

### 3.5 Role of precursor selection, heteroatom doping, and surface functional groups

The role of precursor selection, heteroatom doping, and surface functional groups is fundamental to determining the structural, optical, and sensing properties of CDs. Precursor selection controls the carbonization degree, particle size, surface chemistry, and types of emissive states because different precursors provide different carbon skeletons and functional groups.^69–71^ Heteroatom doping with elements such as nitrogen (N), sulfur (S), phosphorus (P), or boron (B) can modify the electronic structure of CDs, introduce new energy levels, enhance fluorescence intensity and quantum yield, and improve the interaction between CDs and target analytes.^72^ Surface functional groups, including –OH, –COOH, –NH_2_, C=O, and other nitrogen- or sulfur-containing groups, provide active binding sites and determine the surface charge, hydrophilicity, selectivity, and recognition ability of the CDs.^73,74^ Consequently, CDs with different structures can exhibit substantially different sensing performances because variations in particle size, graphitization, defect density, surface states, heteroatom distribution, and functional-group composition alter their emission behavior and analyte–CD interactions.^75,76^ These structural differences can enhance mechanisms such as PET, FRET, IFE, static/dynamic quenching, and aggregation-induced emission, resulting in differences in sensitivity, selectivity, detection limit, and response time. Therefore, rational control of precursor composition, doping level, and surface functionalization is essential for designing CDs with optimized sensing performance for specific analytes.

## 4. Sample preparation for the analysis of pharmaceutical residues in food samples

Food matrices are often highly complex, making sample pretreatment an essential step for achieving accurate and reliable analyte detection.^77^ Sample preparation generally involves physical or chemical procedures designed to separate the target analytes from co-extracted matrix components and/or enrich their concentration in the extract prior to instrumental analysis. Based on this principle, most conventional and officially recognized analytical methods incorporate specific sample-preparation procedures to minimize matrix effects and improve the accuracy and reliability of the analytical results.^78^

This section discusses sample-treatment procedures for the determination of pharmaceutical residues in various food samples, including milk, egg, salmon, pork, and honey. For example, Zhang *et al.* developed CDs for the determination of TC in salmon samples. The practical applicability of the probe was demonstrated using real samples. The salmon sample was homogenized using a juicer, followed by protein precipitation and removal using acetonitrile. Ultrasonication was then performed to facilitate the separation of the precipitate from the solution. Finally, the standard addition method was applied to evaluate the spike recovery of TC.^79^ In addition, Li *et al.* developed novel CDs for the determination of TC in honey samples. Their study focused on the pretreatment of real honey samples. The honey samples were diluted with phosphate-buffered saline (PBS), followed by centrifugation to remove solid components. The resulting supernatant was then collected and used for the determination of TC in the honey samples.^80^ Furthermore, Wang *et al.* developed a CD-based sensor for the determination of TC and ofloxacin in milk and egg samples. For sample pretreatment, the samples were mixed with TA buffer solution, followed by sonication and centrifugation to remove solid components. The resulting solution was then filtered and stored for subsequent analysis.^81^ Similar to, Li *et al.* applied CDs as a sensor for the detection of TC, oxytetracycline, and CTC in real pork samples. The pork samples were mixed with 2% lead acetate and shaken for several minutes, followed by centrifugation. The supernatant was then collected and used for the determination of the target analytes. The standard addition method was employed to evaluate the spike recovery.^82^ Lastly, Guo *et al.* used copper-doped CDs for the determination of TC in milk samples. The milk samples were treated with acetonitrile and subjected to sonication, followed by centrifugation and filtration to remove precipitated and solid components. The resulting liquid phase was then collected and used for the determination of TC.^83^

## 5. Applications

### 5.1 Tetracyclines (TCs)

Tetracyclines (TCs), including tetracycline, chlortetracycline, doxycycline, oxytetracycline, and tigecycline, are broad-spectrum antibiotics extensively applied in livestock and aquaculture for growth promotion and disease prevention.^84^ Misuse and improper disposal lead to their persistence in the environment and food, causing bioaccumulation. Long-term exposure can trigger bacterial resistance, gut imbalance, teeth discoloration, and kidney or liver damage, posing health risks.^85^ Therefore, developing effective analytical methods for TCs, such as high-performance techniques, is essential.

#### 5.1.1 Tetracycline

The work presented eco-friendly blue-emissive CDs for TC detection *via* static quenching coupled with the IFE. The method showed a detection range of 0–80 µM and a LOD of 26 nM, and it was applied to two real samples: milk and egg.^86^ CDs were synthesized from red beet pigment for TC detection based on PET and IFE mechanisms, showing linear ranges of 0.5–30 µM and 30–90 µM, respectively. The LOD was 0.36 µM, and the method was applied to several food samples, including milk, egg, and chicken.^87^

Thiazole-functionalized CDs were used as a sensor for TC *via* the IFE, with a detection range of 0–18 µM and a LOD of 0.7 nM in milk.^88^ Another work presents a fluorescent sensor based on CDs and MOFs for the detection of TC residues in honey samples *via* the IFE. The method showed a LOD of 8.6 nM with a working range of 0.05–8 µM. It was also applied in visual mode, with a working range of 0.25–20 µM and an LOD of 79 nM, as illustrated in Fig. 1.^89^

**Fig. 1:**
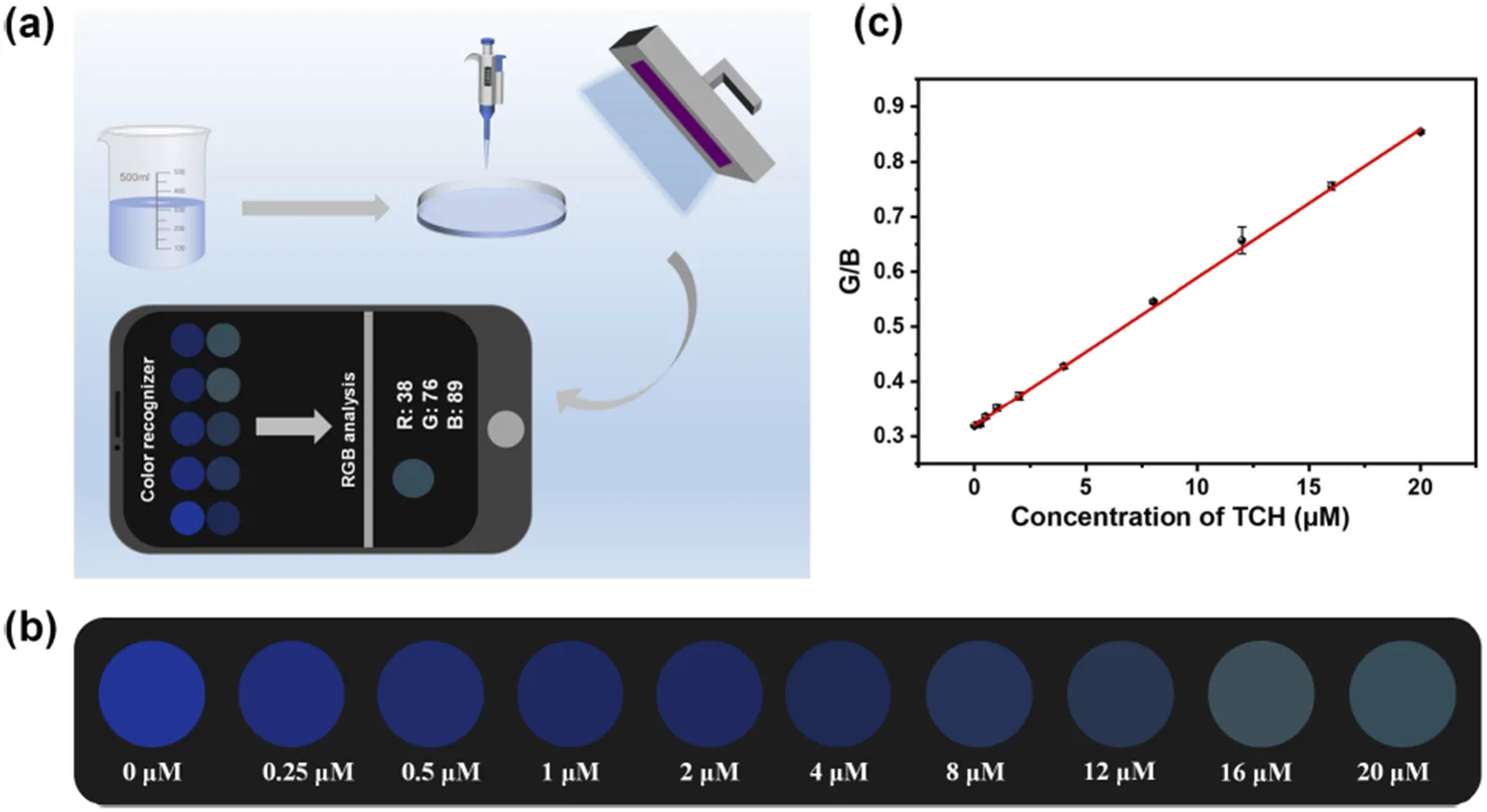
(a) Sensing strategy of the CDs/MOF smartphone-assisted agar platform for TCH detection. (b) Smartphone-monitored color change of agar slices. (c) Linear G/B value *vs.* TCH concentration, reproduced with permission from ref. 89. Copyright 2025, Elsevier.

#### 5.1.2 Chlortetracycline

Red fluorescent carbon dots (R-CDs) synthesized from *Stellaria pallida* and urea were developed as a ratiometric fluorescent probe and incorporated into a portable dual-mode sensing system for detecting CTC residues in food samples. The ratiometric sensor exhibited LODs of 13.00 × 10^−3^ µM for CTC, while the visual sensing platform showed LODs of 1.50 × 10^−3^ µM.^90^ Metal-doped CDs with blue emission at 441 nm were developed for CTC detection, with a working range of 1.0–60 µM and a LOD of 50 nM in milk and bovine serum samples,^91^ as illustrated in Fig. 2.

**Fig. 2:**
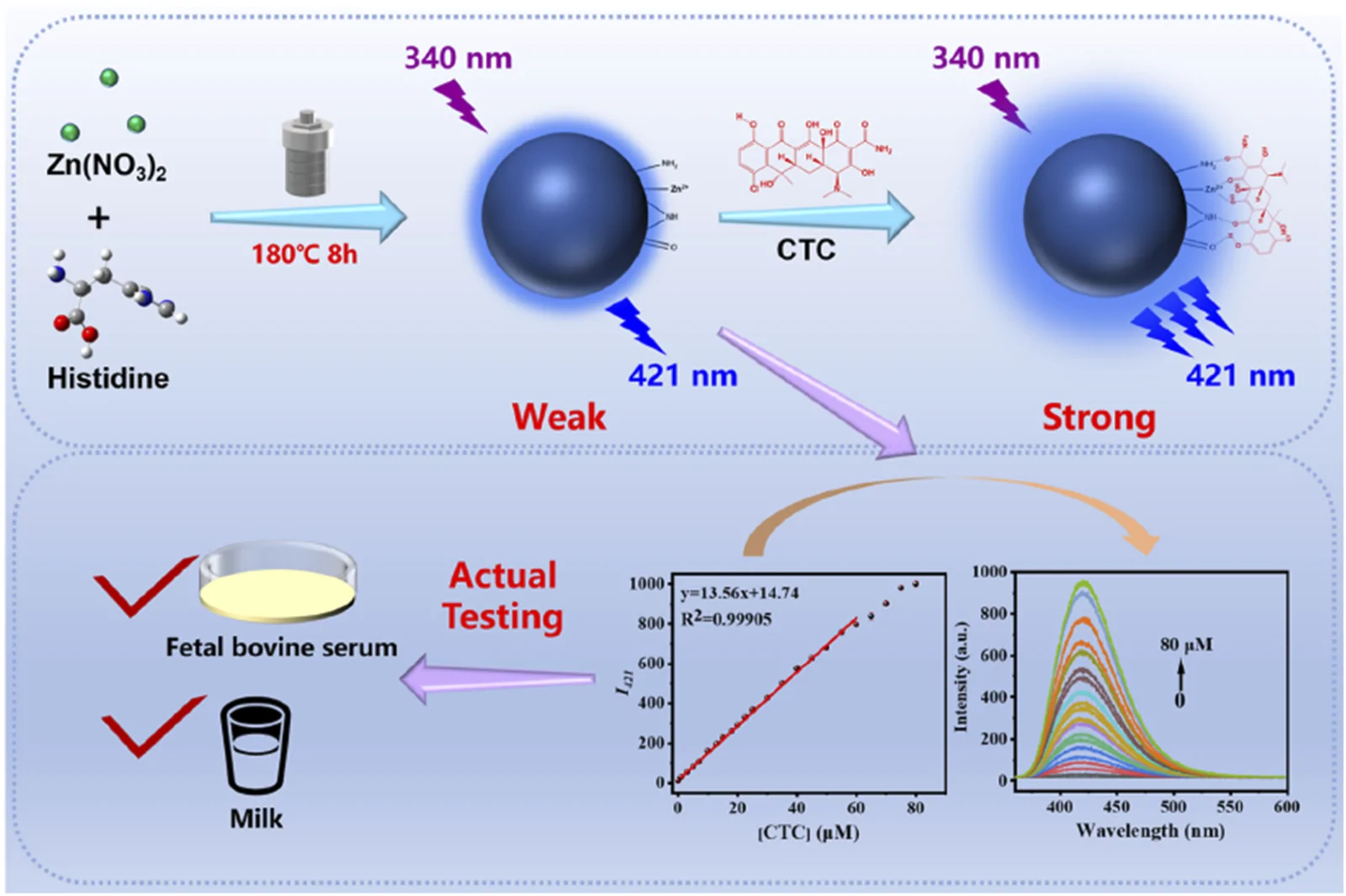
Schematic diagram of the preparation and application of metal-CDs, reproduced with permission from ref. 91. Copyright 2025, Elsevier.

#### 5.1.3 Doxycycline

In this subsection, several studies are discussed on the detection of doxycycline (DOX) *via* the IFE using cerium-doped carbon dots synthesized from ethylenediamine, citric acid, and cerium nitrate. The CDs showed a QY of 93.14%, a LOD of 0.25 µM, and a working range of 0.4–35 µM. The method was applied to milk and pork samples.^92^ The presented work determined DOX using CDs in nanocomposite materials, showing a working range of 0.05–2 mg L^−1^ and a LOD of 3.86 ng mL^−1^ in real food samples.^93^ Dual-emissive CDs were developed for DOX detection *via* IFE in food samples, with a working range of 0.061–80 µM and a LOD of 61 nM.^94^ Lastly, this novel work for DOX detection was developed based on metal-doped CDs, which exhibit two emission peaks at 440 nm and 613 nm. The method shows a LOD of 43 nM and a detection range of 0.1–30 µM in real food samples (milk and honey), with a sensing mechanism based on IFE.^95^

#### 5.1.4 Multiple TCs analysis

Carbon dots were applied for the detection of TC residues, including CTC, OTC, and TC, based on the IFE. The obtained LODs were 0.18 µM for CTC, 0.15 µM for OTC, and 0.13 µM for TC, with milk used as a real sample.^96^ Because doped CDs have attracted considerable research interest, copper-doped CDs were developed here for the detection of TCs through the IFE. In the presence of four tetracycline antibiotics DOX, CTC, TC, and OTC the fluorescence intensity of N, Cu-CDs is quenched. Using a single-signal fluorescence sensing approach, the LODs were 43.8 nM for OTC, 23.8 nM for DOX, 37.2 nM for TC, and 28.8 nM for CTC.^97^ Another type of metal-doped CDs, using europium (Eu) as the dopant, was developed for detecting four tetracycline antibiotics: OTC, TC, DC, and CTC. The linear detection ranges were 0.00–603.75 µM for OTC, 0.00–623.82 µM for TC, 0.00–601.54 µM for CTC, and 0.00–594.61 µM for DC. The corresponding limits of detection were 9.50 nM, 15.80 nM, 90.30 nM, and 10.40 nM, respectively. In addition, a visual detection mode was also implemented for the same purpose.^98^

The research articles on the determination of different types of tetracyclines are extensive and have attracted considerable research interest; therefore, all relevant articles are summarized in Table 1.

**Table 1:** Summary of articles on the determination of tetracyclines using CDs

Antibiotic	Emission color	Sensing mechanism	Working range	LOD	Sample	Ref.
TC, DOX, OTC	Blue, red	IFE	0–60 µM	0.25, 0.39, 0.26 µM	Fish, pork	99
CTC, OTC, MOC, SMX, SMM, SMD	Blue, red	IFE, PET	0.1–100 µM	33, 49, 34, 60, 66, 63 nM	Egg, shrimp, pork, clam, bacon, fish beef crayfish, chicken	100
TC	Blue	IFE, static quenching	0–100 µM	37 nM	Pork, egg, milk, beef	101
DOX, TC, OTC, CTC	Blue	IFE	0.08–3.05, 0.32–5.85, 0.31–6.29, 0.30–6.06 µM	2.87, 14.57, 16.99, 17.11 nM	Milk	102
TC, NOR	Blue	IFE	0–33 µM, 0–65 µM	0.15 µM and 0.87 µM	Milk, soil	103
TC	Blue	IFE	0.1–4.0 µg mL^−1^	0.045 µg mL^−1^	Milk, egg	104
DOX, tigecycline, TC	Blue	—	2–50 ng mL^−1^	2–50 ng mL^−1^	Milk	105
TC	Blue	—	20 nM to 14 µM	5.48 nM	Milk	106
TC, OTC, CTC	Red	IFE	3–40 µM, 4–50 µM, 2–50 µM	38.5 nM, 64.6 nM, 45.4 nM	Milk	107
TC	Red	IFE	0.5–130 µM	0.16 µM	Milk, honey, tea	108
TC	Blue	IFE, PET	0.2–70 µM	0.072 µM	Milk	51
TC	Blue	IFE, static quenching	0.5–60 µM	0.39 µM	Milk	109
TC	Blue	Dynamic, static quenching	0.01–40 µM	14 nM	Milk	110
TC, OTC, CTC	Blue	IFE	0.344–0.265 µM	0.117 µM	Milk	111
TC, OTC, DOX	Blue, green	IFE	0.5–60 µM, 0.5–55 µM, 0.5–60 µM	6.56 nM, 29.46 nM, 30.58 nM	Milk	112
OTC, TC	Blue, red	IFE, static quenching	0.1–45 µM, 0.1–30 µM	0.017, 0.041 µM	Milk, honey	113
TC	Blue	IFE	10–100 µM	0.219 µM	White shrimp	114
TC	Blue, red	IFE	25 nM–20 µM	8.7 nM	Milk	115
TC	Blue	—	40–500 ng mL^−1^	45 ng mL^−1^	Milk	116
TC	Blue	IFE	0–350 µM	0.17 µM	Milk	117
TC, OTC, CTC	Blue	IFE, static quenching, electrostatic interaction	1–60 µM, 1–40 µM, 1–80 µM	—	Honey, milk, fish pork	118
TC	Blue	—	0.106–5 µM	0.032 µM	Milk	119
TC	Blue	IFE	0.1–50 µM	61.1 nM	Milk	120
TC	Blue	IFE	10–100 µM	0.05–0.83 µM	Milk, fresh milk, eggs, chicken, honey	121
TC	Blue	π–π interactions, electrostatic attraction, host–guest interaction	0.01–50 µM	5.04 nM	Milk	122
TC	Blue	IFE	2.94–4.46 ng mL^−1^	3.11 ng mL^−1^	Chicken meat, egg, milk, honey	123
TC	Blue	IFE, static quenching	0–60 µM	96 nM	Milk, egg	124
TC, CTC, OTC	Red	AIEE	3–35, 5–50, 2–50 µM	12, 25, 23 nM	Milk	125
TC	Blue	IFE	5–100 ng mL^−1^	0.79 ng mL^−1^	Milk, eggs, pork	126
TC	Blue	IFE	0–100 µM	13.2 nM	Milk	127
TC, DOC, CTC	Blue	IFE	0–50, 0–50, 0–45 µM	0.025, 0.033, 0.027 µM	Honey, milk	128
TC	Blue	IFE	0.05–30 µM	8 nM	Fish, beef, lamb, pork, egg, chicken, milk, honey	129
TC	Blue, red	IFE	25–275 µM	78 µM, 6.18 µM	Milk	130
TC	Blue	IFE	0.1–65 µM	0.04 µM	Milk	131
TC	Blue	IFE	0.1–50 µM	41.7 nM	Milk	132
TC, OTC, CTC	Blue	FRET	0.5–500 µM, 2–400 µM	0.19, 0.98, 0.36 µM	Milk, honey	133
TC	Blue	IFE	0.1–50 µM	22.6 nM	Milk	134
TC	Blue	IFE	0.4–20 µg mL^−1^	0.073 µg mL^−1^	Pork	135
TC	Blue	IFE	1.88–60 µM	0.56 µM	Milk, honey	136
TC	Green	Static quenching	0.1–10 µM	0.0555 µM	Milk	137
TC	Blue	IFE, static quenching	0.1–500 µM	0.032 µM	Milk, honey	138
TC	Green	IFE, static quenching	0.05–10 µM	16.8 nM	Milk, honey	139
TC	Blue	IFE	0.01–240 µM	3.83 nM	Milk	140
TC	Blue	Static quenching	0.05–45 µM	5.92 nM	Milk, honey	141
TC, OTC, CTC, DOX	Green	IFE	0–100 µM	32.8 nM, 23.3 nM, 41.2 nM, 37.8 nM	Pork, milk, apple	142
DOX	Blue	IFE	0.5–50 µg L^−1^	0.34 nM	Milk, fish, pork, beef, eggs	143
TC	Blue	IFE	4.762–90.91 nM	0.9284 nM	Milk	60
TC	Red	IFE	1.24–165 µM	32.36 nM	Milk	61
TC	Blue	IFE	0–100 µM	0.045 µM	Milk	62
TC	Blue	IFE	2.5 × 10^−10^ to 5 × 10^−6^ M	9.735 × 10^−13^ M	Milk, honey	144
TC	Blue	IEF, charge transfer	0.5–30 mg mL^−1^	0.054 mg mL^−1^	Milk	145
CTC, TET, OCT	Blue	IFE	0–100 µM	0.15, 0.18, 0.12 µM	Milk	146
OTC, TC	Green	IFE, static quenching	0.00–805.20 µM, 0.00–1039.50 µM	42.90 nM, 83.30 nM	Pork, fish	55
TC	Blue	IFE	0–16 µM	33 nM	Milk	147
TC, CTC, DOX, OTC	—	IFE	1–300 µM	0.31, 0.37, 0.30, 0.36 µM	Milk	148
TC	Blue	Dynamic quenching	0–140 µM	0.846 µM	Milk, egg	149

### 5.2 Chloramphenicol

Chloramphenicol (CAP) is extensively applied in the treatment and prevention of infections in livestock and poultry due to its broad-spectrum activity against both Gram-positive and Gram-negative bacteria.^150,151^ However, residual traces of this antibiotic can persist in animal-derived products such as meat and milk, potentially leading to allergic responses, the development of antimicrobial resistance, and suppression of the immune system.^152^

A method was developed for the simultaneous determination of amantadine (AMD) and chloramphenicol (CAP) residues based on FRET between hapten-labeled CDs and antibody-functionalized WS_2_ nanosheets. The proposed immunoassay enabled the concurrent detection of AMD and CAP, achieving detection limits of 0.10 ng g^−1^ and 0.06 ng g^−1^, respectively.^153^ This work was developed based on molecularly imprinted polymers (MIP) and doped CDs for the detection of CAP *via* the IFE. The LOD was 7.65 nM, and the working range was 0.24–26.1 µM.^154^ Another study utilized CDs for the detection of the same drug residue in food samples (milk), achieving an LOD of 0.31 µM and a working range of 0.1–1.0 µM.^155^ A similar approach based on MIP and CDs, combined with blue-emitting CDs and a yellow-emissive dye as a fluorescent probe, was developed for CAP detection in milk samples, with a working range of 0.1–3 µg L^−1^ and an LOD of 0.035 µg L^−1^ (ref. 156) as shown in Fig. 3.

**Fig. 3:**
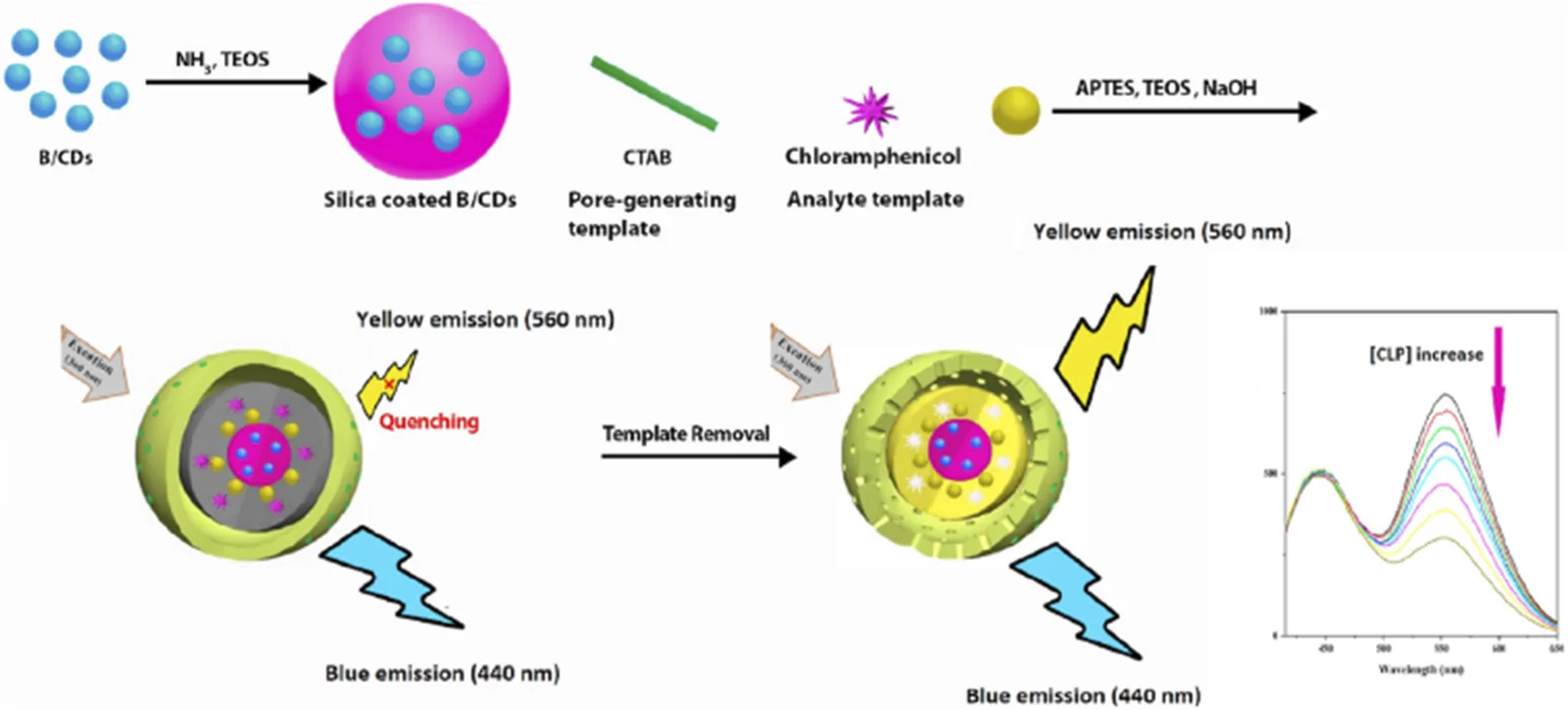
Synthesis and application of mMIP@YBCDs for CAP sensing, reproduced with permission from ref. 156. Copyright 2020, Elsevier.

The research articles on the determination of different chloramphenicol antibiotics have attracted considerable interest; therefore, all relevant articles are summarized in Table 2.

**Table 2:** Summary of articles on the determination of chloramphenicol antibiotics using CDs

Antibiotic	Emission color	Sensing mechanism	Working range	LOD	Sample	Ref.
CAP	Blue	—	0–10 µg kg^−1^	0.03 µg kg^−1^	Chicken	157
CAP	Blue	Fluorescence quenching	0.323–8075.0 µg L^−1^	0.08 µg L^−1^	Milk, honey, pork	158
CAP	Blue	ICT	0.8–70 µM	0.36 µM	Milk	159
CAP	Blue	Static quenching	0.00080–0.0050 mg mL^−1^	0.00030 mg mL^−1^	Milk	160
CAP	Blue	π–π stacking	0.0025–0.02 mg mL^−1^	0.0018 mg mL^−1^	Chicken	161
CAP	Blue	PET	2–80 µM	44 nM	Milk	162

### 5.3 Ciprofloxacin (CIP)

The detection of pharmaceutical residues in food samples based on CDs: in this work, metal-doped CDs were used for the detection of fluoroquinolones (FQs), including difloxacin, ciprofloxacin, and enrofloxacin. The working concentration was 0.01 µM, and the LODs were 7.8 nM, 7.4 nM, and 9.2 nM, respectively, based on the AIE effect and the antenna effect.^163^ The developed sensor for ciprofloxacin (CIP), based on phosphorus-doped CDs, was applied to real milk samples, showing a detection range of 0.01–0.1 µM and a LOD of 6.37 × 10^−3^ µM. It also demonstrated antibacterial efficacy.^164^ Metal-doped CDs were developed for the detection of CIP, exhibiting emission at 670 nm *via* both AIEE and FRET processes, with a detection range of 0.008–120 µM and a LOD of 1.6 nM.^165^ This developed sensor is based on a dual-emission system of CuAg NCs and CDs for CIP detection, with a LOD of 3.3 µM and detection range of 5.0–200.0 µM. The assay can be performed using a smartphone. Smartphone as a detector combined with test strips can be applied to determine CIP concentrations, with a detection range of 0.0–200.0 µM.^166^ MIP and CDs were developed for the detection of CIP in milk samples, showing a detection range of 0.025–6.000 µg mL^−1^ and a LOD of 6.858 ng mL^−1^. Additionally, a portable optosensing platform was employed, covering concentration ranges of 0.080–6.000 µg mL^−1^. Furthermore, the LOD was 22.382 ng mL^−1^,^167^ as shown in Fig. 4.

**Fig. 4:**
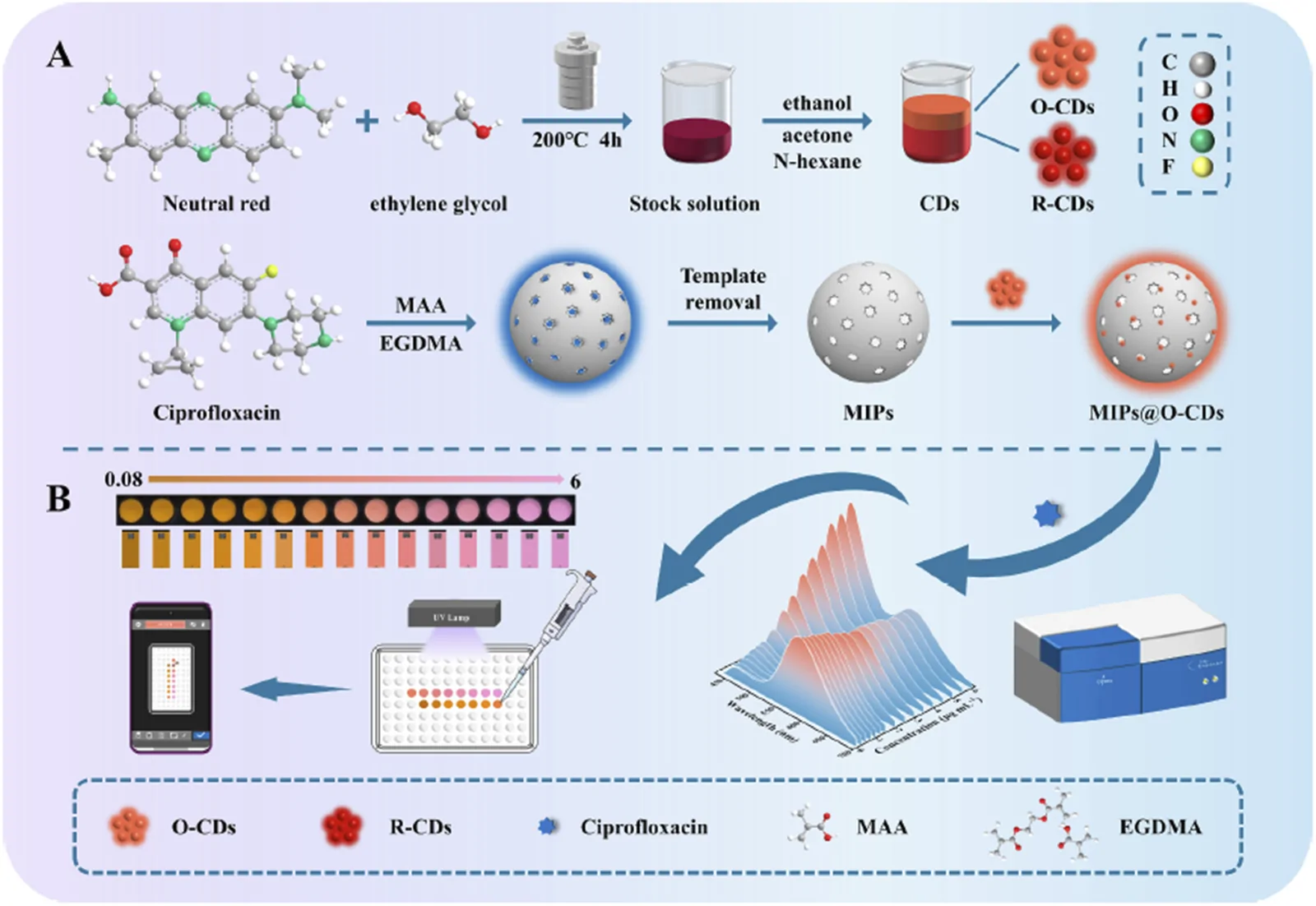
(A) Development of a fluorescence sensing system based on CDs. (B) Application of the probe, reproduced with permission from ref. 167. Copyright 2025, Elsevier.

The research articles on the determination of different fluoroquinolones antibiotics have attracted considerable interest; therefore, all relevant articles are summarized in Table 3.

**Table 3:** Summary of articles on the determination of fluoroquinolones using CDs

Antibiotic	Emission color	Sensing mechanism	Working range	LOD	Sample	Ref.
Difloxacin	Blue	FRET, IFE	0–2.5 × 10^−3^ M	1.77 × 10^−5^ M	Milk	168
Ciprofloxacin	Red	—	5.0–215.0 µM	0.014 µM	Fish	169
Ciprofloxacin	Blue	Intermolecular hydrogen bonds	0.5–25 µM	24.2 nM	Milk, malatang	170
Ciprofloxacin	Blue	IFE	0.05–5.00 µM	4.20 nM	Milk	171
Ciprofloxacin	Green	PET	0.059–20 µM	59 nM	Meat	172
Ciprofloxacin	Blue	—	0–150 µM	0.37 µM	Milk	173
Ciprofloxacin	Blue	IFE, static quenching	0.49–50.10 µM	0.14 µM	Milk	174
Ciprofloxacin	Blue	—	0–100 µM	0.56 µM	Milk	175
Ciprofloxacin	Red	Electrostatic interaction	0.5–800 ng mL^−1^	0.167 ng mL^−1^	Milk	176
Ciprofloxacin	Blue	Electrostatic interaction	0–0.4 µM		Yogurt	177
NOR, ciprofloxacin	Blue	Hydrogen bond interaction	0.02–1.25 µM, 0.02–1.0 µM	4.6 nM, 6.7 nM	Milk	178
Ciprofloxacin	Blue	Hydrogen bonding, electron delocalization	0.1–1 µM	316 nM	Honey	179
CIP	Blue green	IFE, antenna effect	0.5–20 µM, 20–58 µM	—	Milk, fish, beef	180
Ciprofloxacin	Blue, green	FRET	0.06–10 µM	0.2 ng mL^−1^	Grass carp	181
Ofloxacin (OFL)	Blue	Hydrogen bonding and charge transfer promote	0.075–3.75 µM	0.05 µM	Milk	182
Enrofloxacin	Blue	IFE	0–400 µM	—	Honey, milk, eggs, pork	183
Moxifloxacin gatifloxacin, ofloxacin	Green	FRET	0.249–249, 0.266–266, 0.277–277 µM	1.3, 3.2, 5.2 µM	Milk	184
Fluoroquinolones	Green	Electron transfer	5–900 µM	4.40 µM	Milk, honey	185

### 5.4 Gentamicin (GEN)

Gentamicin (GEN), an aminoglycoside antibiotic,^186^ exerts its antibacterial activity by disrupting bacterial protein synthesis and is widely used to treat infections, particularly those caused by Gram-negative bacteria.^187^ In livestock and agricultural practices, antibiotics are commonly incorporated into animal feed to manage infections in cattle, pigs, and poultry. As a result, residues of these drugs can persist in products such as milk, eggs, and meat, leading to human exposure through consumption. However, excessive intake of gentamicin may result in adverse effects, including hearing impairment, nephrotoxicity, and allergic reactions.^188^

Long *et al.* published a study on the determination of GEN using green-emissive CDs in milk samples. The figures of merit, including the working range and LOD, were 12.5–62.5 µM and 0.42 µM, respectively. The sensing mechanism was attributed to static quenching.^189^ Sajwan *et al.* developed another probe for the same drug based on GQDs and AuNPs for use in both milk and egg samples. Due to an IFE mechanism, the method showed a working range of 1.03–16.55 µM, with LODs of 0.422 µM (colorimetric) and 0.493 µM (fluorometric) systems.^190^ Also Sajwan with their team published new research for same drug Guo *et al.* developed a MIP probe for the detection of gentamicin in milk samples, with a working range of 3–900 nM and a LOD of 0.274 nM.^191^ Wei *et al.* developed CDs probe for gentamicin detection, in which a smartphone was used as the detector. The LOD for the colorimetric was 0.30 µM and fluorometric was 14.7 nM.^192^ Guo *et al.* synthesized CDs *via* a one-step hydrothermal method using brown sugar as the precursor. The probe, based on CQDs and AuNPs, exhibited a detection range of 0.56 nM to 5.56 µM and a LOD of 0.56 nM.^193^

### 5.5 Sulfonamides

Sulfonamides (SAs) are broad-spectrum antibacterial agents containing *p*-aminobenzene sulfonamide. They are extensively used worldwide because of their chemical stability, ease of application, and low cost. However, their large-scale production and improper or excessive use have led to significant risks, including food contamination and environmental pollution.^194^

Li *et al.* developed a probe based on CDs prepared from shikimic acid for the determination of sulfasalazine (SSZ). The probe showed a LOD 60 pM and linear range of 0–1 µM.^195^ Yang *et al.* employed metal-doped CDs for the detection of *o*-toluenesulfonamide (TFA) and sulfamethoxazole (STZ). The sensor showed sensitive detection with low LODs of 0.09 µM for TFA and 0.05 µM for STZ, and its applicability in chicken sample analysis was successfully demonstrated.^196^ Similarly, Chen *et al.* employed co-doped CDs for the detection of sulfathiazole (STZ). The fluorescent probe enabled accurate determination of STZ with a low LOD of 5.5 ng mL^−1^ across two wide working ranges of 0.008–10 µg mL^−1^ and 10–45 µg mL^−1^.^197^ Lastly, Cai *et al.* designed a MIP-based ratiometric fluorescence sensor for sulfadiazine (SDZ) using biomass-derived CDs and CDs synthesized from organic molecules. The system achieved a LOD of 10.41 nM, markedly better than the 25.94 nM obtained with a single-channel sensor,^198^ as shown in Fig. 5.

**Fig. 5:**
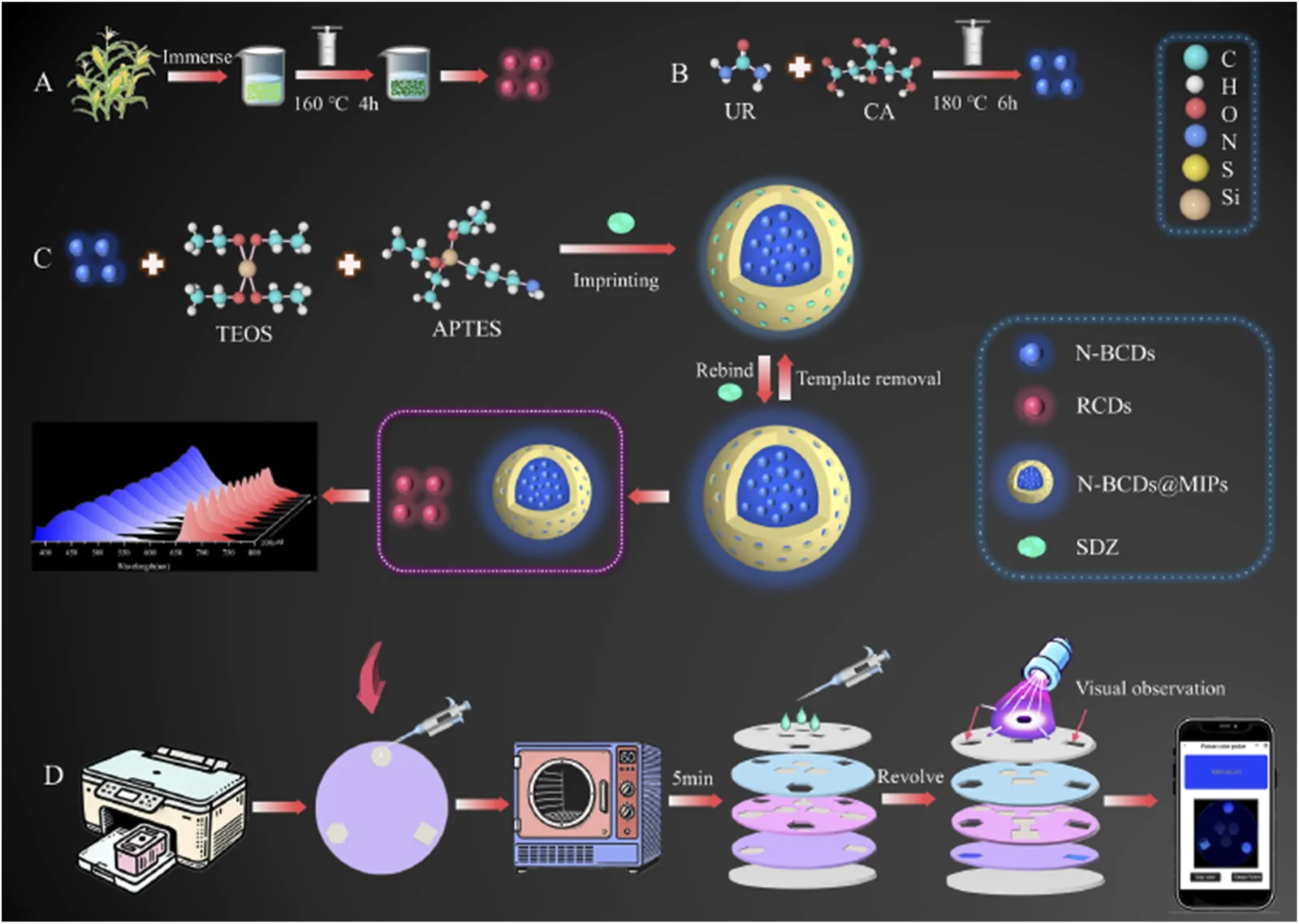
Schematic illustration of the synthesis of (A) biomass-derived CD, (B) nitrogen doped CD, (C) sensor fabrication, and (D) SDZ detection using a rotary microfluidic paper chip, reproduced with permission from ref. 198. Copyright 2025, Elsevier.

### 5.6 Other drugs in food samples

Remaining pharmaceuticals in food samples may pose significant hazards to human health; therefore, the development of new CD-based sensors for this purpose has attracted great interest. Other pharmaceutical residues in food, including cefoperazone, cephalexin ceftriaxone, chlorogenic acid, tinidazole, erythromycin, florfenicol, gatifloxacin, kanamycin, metronidazole, moxifloxacin, sparfloxacin, orbifloxacin, tobramycin, and penicillin G, determined using CDs are summarized in Table 4.

**Table 4:** Summary of articles on the determination of other drugs using CDs

Antibiotic	Emission color	Sensing mechanism	Working range	LOD	Sample	Ref.
Cefoperazone	Green	IFE, static quenching	0.01–500 µM	8 nM	Milk	199
Cephalexin, ceftriaxone	Blue, green	Hydrogen bonding	0.10–50.0 µg L^−1^	0.06 and 0.10 µg L^−1^	Milk	200
Chlorogenic acid (CGA) and tinidazole (TNZ)	Blue	Static quenching, IFE	0.036–40 µM, 0.068–25 µM	36 nM, 68 nM	Milk, coffee	201
Erythromycin	Green	Static quenching	1.93–377.08 µM	0.6693 µM	Milk	202
Florfenicol	Blue	—	0.50–100 µg L^−1^	0.17 µg L^−1^	Milk	203
Gatifloxacin	Green	Hydrogen bond	0.01–100 µM	8 nM	Milk, honey	204
Kanamycin	Blue	—	0.04–0.24 µM	18 nM	Milk	205
Metronidazole	Blue	IFE	0–240 µM	0.19 µM	Milk, honey	206
Metronidazole	Blue	IFE	0.5–22 µM	0.22 µM	Milk	207
Metronidazole	Blue	Static quenching	0.06–15 µg mL^−1^	20 ng mL^−1^	Milk	208
Metronidazole	Blue	IFE	0–10 µg mL^−1^	0.257 µg mL^−1^	Honey	209
Moxifloxacin	Blue, green	Static and dynamic quenching	0.05–0.5 µM	17.5 nM	Milk	210
Moxifloxacin	Green	IFE	0.025–15.0 µM	6.34 nM	Milk, egg	211
Sparfloxacin, orbifloxacin	Blue	Hydrogen bonding, charge-transfer	1.5–130 nM, 1.2–110 nM	0.5 nM, 0.4 nM	Milk	212
Tobramycin	Blue	—	1–12 µM	99.2 nM	Milk	213
Penicillin G	Blue, red	IFE	0.1–0.8 ppm and 0.8–30.0 ppm	30.0 ppb	Milk	214
Penicillin G	Blue, yellow	—	1–32 nM	0.34 nM	Milk	215

## 6. Future perspectives

Despite the rapid development of carbon dot (CD)-based fluorescent probes for pharmaceutical residue detection, their transition from laboratory-based research to routine food-safety monitoring remains limited. Future research should therefore move beyond simply improving fluorescence intensity or detection limits and focus on selectivity, mechanism-driven design, matrix compatibility, portability, validation, and real-world applicability.

(1) Rational design of CDs for target-specific recognition. Future studies should establish clear relationships between precursor selection, particle size, surface functional groups, heteroatom doping, and optical properties. Computational chemistry and machine-learning approaches could assist in predicting CD structures with specific binding interactions toward antibiotics, analgesics, hormones, and other pharmaceutical residues.

(2) Improved understanding of fluorescence quenching mechanisms. Although mechanisms such as the IFE, PET, FRET, dynamic quenching, static quenching, and aggregation-related effects have been widely proposed, they are often not adequately distinguished experimentally. Future work should combine steady-state and time-resolved fluorescence, UV-vis spectroscopy, lifetime measurements, electrochemical analysis, and computational calculations to provide convincing mechanistic evidence.

(3) Development of highly selective recognition strategies. Selectivity remains a major challenge because food samples contain proteins, lipids, pigments, sugars, salts, and other compounds that can interfere with fluorescence measurements. Surface functionalization, molecular imprinting, aptamer integration, antibody-based recognition, and CD–MOF or CD–MIP hybrid systems could provide more selective recognition of structurally similar pharmaceutical compounds.

(4) Ratiometric and multiplex sensing. Future CD probes should increasingly employ ratiometric fluorescence rather than single-intensity measurements because ratiometric signals can reduce the effects of probe concentration, excitation intensity, environmental conditions, and sample matrix. Combining CDs with different emission centers could also enable simultaneous detection of multiple pharmaceutical residues in a single analysis.

(5) Integration with smartphones and portable devices. CD-based probes have strong potential for on-site food-safety monitoring. Future research should develop paper-based, hydrogel, microfluidic, lateral-flow, and smartphone-assisted platforms capable of providing rapid quantitative or semiquantitative results without sophisticated laboratory instrumentation.

(6) Real-food validation and standardized analytical performance. Many reported CD sensors are evaluated mainly in buffer solutions or spiked samples. Future studies should prioritize unprocessed and commercially available food matrices, including milk, meat, fish, eggs, honey, vegetables, and beverages. Recovery, precision, selectivity, stability, matrix effects, and reproducibility should be systematically evaluated and compared with established techniques.

(7) Green and sustainable CD synthesis. The use of food waste, agricultural residues, biomass, and other renewable precursors should be further explored to produce CDs with lower environmental impact. However, future studies should assess batch-to-batch reproducibility, precursor variability, energy consumption, solvent use, purification requirements, and overall environmental footprint, rather than assuming that biomass-derived CDs are automatically green.

(8) Artificial intelligence and data-driven sensing. Machine learning could be combined with fluorescence spectra, RGB images, or multidimensional sensor responses to improve recognition of complex pharmaceutical-residue patterns. This approach could facilitate simultaneous identification and quantification of multiple contaminants in complicated food matrices.

(9) Standardization and commercialization. A major future challenge is the lack of standardized protocols for CD synthesis, fluorescence characterization, quantum-yield determination, quenching-mechanism verification, and analytical validation. Establishing common reporting standards would improve reproducibility and facilitate comparison among published sensors. Long-term stability, storage conditions, sensor shelf life, and large-scale manufacturing should also be addressed before commercial implementation.

(10) Improving batch-to-batch reproducibility. Future research should focus on standardizing precursor selection, synthesis conditions, purification procedures, and surface functionalization of carbon dots to achieve consistent physicochemical and fluorescence properties, thereby improving the reliability, reproducibility, and practical application of CD-based sensors in food analysis.

(11) Correlating reported detection limits with regulatory requirements. If a regulatory limit or maximum residue limit (MRL) exists for the food contaminant, the reported detection limit should be correlated to the regulatory limit. The analytical sensitivity of CD-based sensors can be compared to these regulatory thresholds to assess whether the reported LOD is low enough to ensure reliable food-safety monitoring and practical use.

## 7. Conclusion

Over the past decades, the use of pharmaceuticals in human and veterinary medicine has increased dramatically. In addition to their excessive use, the presence of trace amounts of pharmaceutical compounds in food samples has become a major concern because it may contribute to the development of drug resistance. Therefore, the detection of pharmaceuticals in food samples is of great importance. In conclusion, this review comprehensively discusses the toxicity of pharmaceutical residues in food samples and their effects on human health, as well as their determination using CD-based sensing systems. The sensing mechanisms between CDs and pharmaceutical compounds are also explained, and the applications of CDs for monitoring a wide range of pharmaceuticals are highlighted.

## Conflicts of interest

There are no conflicts to declare.

## Data Availability

There is no additional data associated with this article.
